# Epigenomic profiling of neuroblastoma cell lines

**DOI:** 10.1038/s41597-020-0458-y

**Published:** 2020-04-14

**Authors:** Kristen Upton, Apexa Modi, Khushbu Patel, Nathan M. Kendsersky, Karina L. Conkrite, Robyn T. Sussman, Gregory P. Way, Rebecca N. Adams, Gregory I. Sacks, Paolo Fortina, Sharon J. Diskin, John M. Maris, Jo Lynne Rokita

**Affiliations:** 1 Division of Oncology, Children’s Hospital of Philadelphia, Philadelphia, Pennsylvania 19104 USA; 2 Genomics and Computational Biology Graduate Group, University of Pennsylvania, Philadelphia, PA 19104 USA; 3 Department of Bioinformatics and Health Informatics, Children’s Hospital of Philadelphia, Philadelphia, Pennsylvania 19104 USA; 4 Pharmacology Graduate Group, University of Pennsylvania, Philadelphia, PA 19104 USA; 5 Cancer Genomics and Bioinformatics Laboratory, Sidney Kimmel Cancer Center, Philadelphia, Pennsylvania 19107 USA; 6 Department of Pediatrics, Perelman School of Medicine at the University of Pennsylvania, Philadelphia, Pennsylvania 19104 USA; 7 Center for Data-Driven Discovery in Biomedicine, Children’s Hospital of Philadelphia, Philadelphia, Pennsylvania 19104 USA

**Keywords:** Paediatric cancer, Cancer genomics

## Abstract

Understanding the aberrant transcriptional landscape of neuroblastoma is necessary to provide insight to the underlying influences of the initiation, progression and persistence of this developmental cancer. Here, we present chromatin immunoprecipitation sequencing (ChIP-Seq) data for the oncogenic transcription factors, MYCN and MYC, as well as regulatory histone marks H3K4me1, H3K4me3, H3K27Ac, and H3K27me3 in ten commonly used human neuroblastoma-derived cell line models. In addition, for all of the profiled cell lines we provide ATAC-Seq as a measure of open chromatin. We validate specificity of global MYCN occupancy in MYCN amplified cell lines and functional redundancy of MYC occupancy in MYCN non-amplified cell lines. Finally, we show with H3K27Ac ChIP-Seq that these cell lines retain expression of key neuroblastoma super-enhancers (SE). We anticipate this dataset, coupled with available transcriptomic profiling on the same cell lines, will enable the discovery of novel gene regulatory mechanisms in neuroblastoma.

## Abstract (summary)

**Table:** 

Measurement(s)	histone H3-K4 methylation • histone H3-K27 methylation • histone acetylation • TF_binding_site • open_chromatin_region
Technology Type(s)	ChIP-seq • ChIP-Seq • ATAC-seq
Factor Type(s)	type of neuroblastoma cell line • MYCN amplification vs no MYCN amplification
Sample Characteristic - Organism	Homo sapiens

Machine-accessible metadata file describing the reported data: 10.6084/m9.figshare.11993883

## Background & Summary

An estimated 15,780 children in the United States will be diagnosed with cancer in 2019^1^. While 80% of pediatric cancer patients overcome this disease, 20% of children do not survive, and survivors often have multiple side effects of therapy^1^. Neuroblastoma accounts for more than 7% of malignancies in patients under 15 years of age and approximately 12% of all pediatric cancer-related deaths (for review see^2^). Neuroblastoma shows wide phenotypic variability, with tumors arising in children diagnosed under the age of 18 months often spontaneously regressing with little or no treatment, but patients diagnosed at an older age or with unfavorable genomic features often showing a relentlessly progressive and widely metastatic disease pattern despite intensive, multimodal therapy (for review see^2–4^). Ninety-eight percent of low-risk neuroblastoma disease are currently cured^5^, however, the survival rate for patients with high-risk neuroblastoma remains less than 50%^6^. Relapsed high-risk neuroblastoma is typically incurable^7^, and thus these children require improved therapeutic options.

A major prognostic factor predicting the severity, risk, and inferior outcome for neuroblastoma patients is amplification of the proto-oncogene *MYCN*. *MYCN* amplification occurs in nearly 20% of all neuroblastomas, and approximately 50% of patients with high-risk disease^8,9^. It is a truncal genomic event, and typically stable across the spectrum of therapy and disease recurrence. MYCN, along with structural and binding homologues MYC and MYCL, are members of the MYC transcription factor family^10^ and have been implicated in transcriptional regulation of proteins involved in cell growth^11^, proliferation^12^, and ribosome biogenesis^12^. Mounting evidence has also indicated that MYCN and MYC are functionally redundant^13–15^. However, the protein expression of MYC and MYCN appears to be mutually exclusive. For example, neuroblastoma tumors with *MYCN* amplification typically lack or have low *MYC* mRNA expression^9^. The strong influence of MYCN on the progression and metastasis of neuroblastoma makes it a key target for therapy, but due to its global transcriptional activity, it is necessary to develop a better understanding of which of its gene targets directly influence oncogenesis.

To better understand the regulatory effects of MYC family proteins in neuroblastoma, we performed ChIP-Seq data for MYCN in six neuroblastoma cell lines with *MYCN* amplification, MYC in four neuroblastoma cell lines without *MYCN* amplification, and H3K27Ac, H3K27me3, H3K4me1, and H3K4me3 histone modifications along with ATAC-Seq in all ten neuroblastoma cell lines (with ATAC data in four additional lines also reported here). All of the cell lines here also have RNA sequencing data freely available^16^.

## Methods

**Fig. 1:**
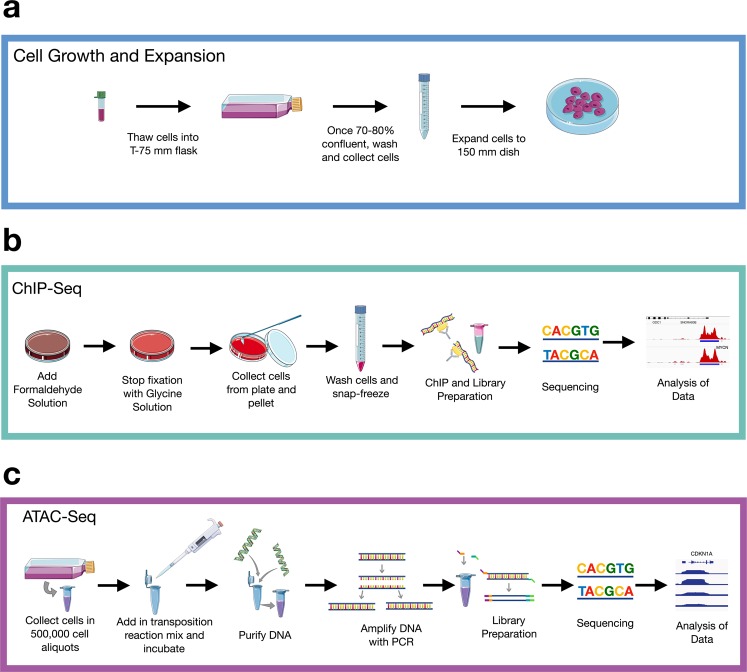
Experimental workflow. (**a**) Cells were thawed, grown, and expanded until 70–80% confluency in a 150 mm dish. (**b**) For ChIP-Seq, cells were fixed, collected, and frozen (N = 1 biological replicate per cell line). Libraries were prepared, sequenced, and data analyzed. (**c**) For ATAC-Seq (n = 14 samples with n = 2 biological replicates), cells were incubated in a transposition reaction, DNA was purified, and amplified with limited PCR. Libraries were prepared, sequenced, and analyzed. Diagram was created using Servier Medical ART (https://smart.servier.com/).

Online Table 1 summarizes which assays were performed for each cell line, and an overview of the workflow is shown in Fig. 1.

### Cell growth and expansion

The cell lines used to collect this data were obtained from multiple sources: the Children’s Oncology Group (COG) Cell Culture and Xenograft Repository at Texas Tech University Health Sciences Center (www.cccells.org), the American Type Culture Collection (Manassas, MA), or the Children’s Hospital of Philadelphia (CHOP) cell line bank. All of the cell growth and preparations were done at CHOP. The neuroblastoma cell lines were cultured using media (Table 1) and methods as previously described^16^. Briefly, cells were thawed by floating in a 37 °C water bath for 2–3 minutes. Cells were then added to a 15 mL conical tube, containing 5 mL of the appropriate growth media, and centrifuged at 300 × *g* for 5 minutes at room temperature (RT). Media was then carefully aspirated off, and the pelleted cells were resuspended in 1 mL of media before being transferred to a 75 mm2 flask containing 10 mL of growth media. Cells were incubated at 37 °C with a 5.0% CO_2_ concentration. When cells reach 70–80% confluency, media was aspirated off and cells were gently washed with 1X PBS. Following aspiration of the PBS, 3 mL of the appropriate detachment solution (noted in Table 1) was added and the flask was incubated at 37 °C for 2–5 minutes. Cells were then gathered by tilting the plate at a 45° angle and washing with at least 4 mL of the appropriate growth media, and transferred to a 15 mL conical. After centrifugation for 5 minutes at 300 × *g*. Media was aspirated off, and the pellet was resuspended in 1 mL of growth media and transferred to a 150 mm cell culture dish containing 19 mL of growth media. Cells were incubated at 37 °C with a 5.0% CO_2_ concentration until reaching 70–80% confluency. Necessary materials and reagents are listed in Online Table 2.

**Table 1:** Neuroblastoma cell line information.

Cell Line	Organism	Cancer Histology	Matched PDX	MYCN Status	Growth Media	Detachment Method
COG-N-415	Homo sapiens	Neuroblastoma	COG-N-415x	Amplified	IMDM, 20% FBS, 2 mM L-glutamine, 1:1000 ITS Premix Supplement	0.02% Versene
KELLY	Homo sapiens	Neuroblastoma	N/A	Amplified	RPMI 1640, 10% FBS, 1% Penicillin/Streptomycin, 2 mM L-Glutamine	0.02% Versene
LA-N-5	Homo sapiens	Neuroblastoma	N/A	Amplified	RPMI 1640, 10% FBS, 1% Penicillin/Streptomycin, 2 mM L-Glutamine	0.02% Versene
NB-1643	Homo sapiens	Neuroblastoma	NB-1643	Amplified	IMDM, 20% FBS, 1% Penicillin/Streptomycin, 2 mM L-glutamine	0.02% Versene
NB-69	Homo sapiens	Neuroblastoma	N/A	Non-Amplified	RPMI 1640, 10% FBS, 1% Penicillin/Streptomycin, 2 mM L-Glutamine	0.02% Versene
NB-LS	Homo sapiens	Neuroblastoma	N/A	Non-Amplified	RPMI 1640, 10% FBS, 1% Penicillin/Streptomycin, 2 mM L-Glutamine	0.02% Versene
NGP	Homo sapiens	Neuroblastoma	N/A	Amplified	RPMI 1640, 10% FBS, 1% Penicillin/Streptomycin, 2 mM L-Glutamine	0.02% Versene
SK-N-AS	Homo sapiens	Neuroblastoma	SK-N-AS (xenograft)	Non-Amplified	RPMI 1640, 10% FBS, 1% Penicillin/Streptomycin, 2 mM L-Glutamine	0.05% Trypsin/EDTA
SK-N-BE(2)-C	Homo sapiens	Neuroblastoma	N/A	Amplified	RPMI 1640, 10% FBS, 1% Penicillin/Streptomycin, 2 mM L-Glutamine	0.02% Versene
SK-N-FI	Homo sapiens	Neuroblastoma	N/A	Non-Amplified	RPMI 1640, 10% FBS, 1% Penicillin/Streptomycin, 2 mM L-Glutamine	0.02% Versene
SK-N-SH	Homo sapiens	Neuroblastoma	N/A	Non-Amplified	RPMI 1640, 10% FBS, 1% Penicillin/Streptomycin, 2 mM L-Glutamine	0.05% Trypsin/EDTA

Listed are the cell lines used in this study, their MYCN amplification status, and culturing media information.

### Immunoblotting

Whole cell lysates were prepared using a mixture of cell lysis buffer (Cell Signaling, #9803), PSMF (Cell Signaling, 8553S), Phosphatase Inhibitor Cocktail 2 (P5726, Sigma Aldrich), Phosphatase Inhibitor Cocktail 3 (P0044, Sigma Aldrich), and PBS (Gibco, 14190–136). Cells were resuspended in lysis buffer and kept on ice for 15 minutes. Cells were then spun at *14,000* × *g* at 4 °C for 15 minutes. The supernatant was collected and protein concentration was quantified using the Pierce BCA Protein Assay kit (Thermo Scientific, #23225). Next, 20 μg of protein was loaded using 4X Laemmli sample buffer (BioRad, #1610747) and separated on a 4–15% Criterion™ TGX™ Precast Midi Protein Gel (#5671085), and transferred to an Immobilon Membrane (Cat No. IPVH00010, 0.45 μm pore size). The membrane was blocked in 5% non-fat milk in Tris-buffered saline and Tween-20 (TBS-T) at room temperature for one hour. Incubation with primary antibody was overnight, rocking at 4 °C. Membranes were then washed three times for 10 mins in TBS-T, and then incubated with HRP-labeled Rabbit secondary antibody at room temperature for one hour (1:2000–1:5000; Millipore, AP132P). The membranes were then developed using chemiluminescence (SuperSignal West Femto, Thermo Fischer Scientific). The primary antibodies used were: N-MYC (1:1000; Cell Signaling, #9405S), MYC (1:800; Cell Signaling #5605), and β-Actin (1:5000; Cell Signaling, #4967S).

### ChIP-Seq protocol

The ChIP-Seq Protocol is separated into four sections: Cell Fixation, Chromatin Immunoprecipitation (ChIP) and Library Preparation, Library Sequencing, and ChIP-Seq Analysis. Of note, the MYCN ChIP-Seq for Kelly and NGP cell lines was performed using a varied procedure and is noted in a separate section within this protocol. Necessary materials and reagents are listed in Online Table 2.

#### Cell fixation

Cells were grown as described in Cell Growth and Expansion section of protocol to 70–80% confluence in 150 mm tissue culture plates in 20 mL of media. The Formaldehyde solution (Online Table 2) was freshly prepared. Cells were removed from incubation and 1/10th of the growth media volume of the Formaldehyde Solution was added to the existing media in the plate (i.e. if the current volume of the plate is 20 mL of media, 2 mL of Formaldehyde Solution would be added). The solution was gently swirled, and then rocked at RT for 15 minutes. To stop the fixation, 1/20th the current volume of the Glycine Solution (Online Table 2) was added to the plate (i.e. if the current volume in the plate is 22 mL then 1.1 mL of Glycine Solution should be added). The plate was gently swirled to mix, and then allowed to sit at RT for 5 minutes. Following this incubation, a cell scraper was used to collect the cells, and then all cells and solution were transferred to a 50 mL conical on ice. From this point forward, all samples were kept on ice. The 50 mL conical was centrifuged at 800 × *g* at 4 °C for 10 minutes to pellet the cells. Supernatant was removed and discarded, and the cells were resuspended with 10 mL of chilled, sterile PBS. Centrifugation of the tube at 800 × *g* at 4 °C for 10 minutes was repeated. The supernatant was removed and discarded, and the cells were resuspended with 10 mL of chilled, sterile PBS with 100 uL of PMSF. The tube was centrifuged at 800 × *g* at 4 °C for 10 minutes, the supernatant was removed, and then the cells were snap frozen on dry ice and stored at −80 °C. The cells were then shipped to Active Motif on dry ice following the instructions listed at on the Sample Submission Form, downloaded from www.activemotif.com/sample-submission.

#### ChIP and library preparation by active motif

Chromatin immunoprecipitation was completed by Active Motif. Full methods are proprietary. Chromatin was isolated using a lysis buffer and membranes were disrupted with a dounce homogenizer. The lysates were then sonicated with Active Motif’s EpiShear probe sonicator (#53051) and cooled sonication platform (#53080) to an average fragment length 300–500 bp. A portion of the sample was collected as the Input DNA, treated with RNase, proteinase K, and incubated to reverse crosslinking. The DNA was then collected by ethanol precipitation. The Input DNA was resuspended and concentration was quantified by a NanoDrop spectrophotometer. Extrapolation of this concentration to the original chromatin volume allowed for quantitation of the total chromatin yield. Aliquots of the fixed chromatin were used in the immunoprecipitation were precleared with protein A agarose beads (Invitrogen, #15918014). Genomic DNA regions of interest were isolated using specific ChIP antibodies (Online Table 2). Antibody DNA complexes were isolated using additional protein A agarose beads, and the crosslinked DNA, antibody, and bead complexes were washed. The cross-linked DNA was eluted from the beads with SDS buffer, and subjected to RNase and proteinase K treatment. Reverse crosslinking was done in an overnight incubation at 65 °C, and ChIP DNA was purified with a phenol-chloroform extraction and ethanol precipitation.

Illumina sequencing libraries were prepared from the ChIP and Input DNAs using the standard consecutive enzymatic steps of end-polishing, dA-addition, and adaptor ligation using Active Motif’s custom liquid handling robotics pipeline. Samplers were amplified with a 15 cycle PCR amplification and then quantified before being shipped to the Jefferson Cancer Genomics Laboratory at the Kimmel Cancer Center for sequencing.

#### MYCN ChIP-Seq: Kelly and NGP cell lines

Chromatin immunoprecipitation was performed on adherent cells as described in Bosse *et al*.^17^. Of note, a different MYCN antibody was used than listed in Bosse *et al*., 2017 (Santa Cruz B8.4B, sc-53993). Cells were grown as described in Cell Growth and Expansion section of protocol to 70–80% confluence in 150 mm tissue culture plates in 20 mL of media. To the existing media, 415 mL of 37% formaldehyde (final concentration of 0.75%) was added, and rocked for 10 minutes at RT. To this, 1.5 mL of 2.5 M glycine (Online Table 2) (final concentration of 0.18 M) was added to inactivate the formaldehyde, and the plate was rocked for an additional 5 min. Cells were lysed with a volume of FA Lysis Buffer (Online Table 2) equivalent to 5 pellet volumes. Beads were washed 3 times in ChIP Wash Buffer (Online Table 2) and one time with Final Wash Buffer (Online Table 2). Libraries were constructed using NEB Ultra Kit following the manufacturer’s instructions. Libraries were sequenced as single-end, 50 bp reads on a MiSeq to a depth of ~50 M reads by the Children’s Hospital of Philadelphia Nucleic Acid and PCR Core.

#### ChIP library sequencing for ChIP

Sequencing was conducted by the Jefferson Cancer Genomics Laboratory at the Kimmel Cancer Center. Samples were quality control tested using an Agilent High Sensitivity Screen Tape to determine average fragment length. The concentration of each library was measured using a High Sensitivity Qubit Quantification kit, and samples were diluted to an appropriate amount for the loading protocol (4 nM or less). Samples were normalized to the same nanomolar concentration, and libraries were pooled together in equal amounts. Samples were diluted to 1.51 pM in Low EDTA TE Buffer. Samples were then sequenced as single-end, 75 bp reads to an average depth of ~30 M reads on a NextSeq. 500.

### ATAC-Seq protocol

The following ATAC-Seq protocol was adapted from Buenrostro, *et al*.^18^. This protocol consists of four parts: Cell Preparation, Transposition Reaction and Purification, PCR Amplification, qPCR, and Library Preparation. Primer 1 and Primer 2 were custom synthesized by Integrated DNA Technologies (IDT), using sequences provided in Buenrostro, *et al*., 2015. Note: ATAC-Seq for NB-69 and NGP was performed using a slightly varied procedure and is noted in a separate section.

#### Cell preparation

Cells were grown as described in Cell Growth and Expansion section of protocol to 70–80% confluence in a 75 mm^2^ tissue culture flasks in 10 mL of media. Following detachment and pelleting, cells were resuspended in 1.0 mL of the appropriate growth media. Cells were triturated until they were in a homogenous single-cell suspension. Using an automated cell counter, the volume for 500,000 cells was determined and aliquoted into a sterile 1.5 mL Eppendorf tube containing 500 μL of sterile 1X PBS. Cells were centrifuged at 500 × *g* for 5 minutes at 4 °C. The supernatant was carefully aspirated, and the cells were resuspended in 500 mL of sterile 1X PBS. Centrifugation was repeated and cells were resuspended in 500 mL of cold lysis buffer by gently pipetting up and down, and then immediately centrifuged at 500 × *g* for 10 minutes at 4 °C. The supernatant was carefully removed and discarded. The pellet was immediately resuspend in 50 μL of nuclease free water by gently pipetting up and down, and the protocol immediately continued on to Transposition Reaction and Purification section.

#### Transposition reaction and purification

The pellet was placed on ice. The following reagents were prepared and combined: transposition reaction mix (25 μL TD (2X reaction buffer from Nextera Kit), 2.5 μL TDE1 (Nextera Tn5 Transposase from Nextera Kit), 17.5 μL nuclease-free water, and 5.0 μL of resuspended DNA/protein from the final step in *Cell Preparation* (resuspended pellet in 50 μL of nuclease free water). The transposition reaction was incubated in a thermocycler at 37 °C for 30–35 minutes. The reaction was immediately purified using Qiagen MinElute PCR Purification Kit, and the transposed DNA was eluted in 10.5 μL of elution buffer (Buffer EB from the MinElute Kit consisting of 10 mM Tris-Cl (pH 8)). The eppendorf tube containing purified DNA was parafilmed, and stored at −20 °C. *NOTE:* This can act as a good stopping point, however these DNA fragments are not PCR amplifiable if melted at this point.

#### PCR amplification

Primer sequences are shown in Table 2. To amplify the Transposed DNA, the following were combined into a 0.2 mL PCR tube: 10 μL transposed DNA, 10 μL nuclease-free H_2_O, 2.5 μL 25 mM PCR Primer 1 (Ad1), 2.5 μL 25 mM Barcoded PCR Primer 2 (Ad2.X, X being the unique number of samples), and 25 μL NEBNext High-Fidelity 2X PCR Master Mix. The thermal cycle was as follows:

$$\begin{array}{rl}1\,{\rm{c}}{\rm{y}}{\rm{c}}{\rm{l}}{\rm{e}}: & 5\,{\rm{m}}{\rm{i}}{\rm{n}}\,7{2}^{\circ }{\rm{C}}\\  & 30\,{\rm{s}}{\rm{e}}{\rm{c}}\,9{8}^{\circ }{\rm{C}}\\ 5\,{\rm{c}}{\rm{y}}{\rm{c}}{\rm{l}}{\rm{e}}{\rm{s}}: & 10\,{\rm{s}}{\rm{e}}{\rm{c}}\,9{8}^{\circ }{\rm{C}}\\  & 30\,{\rm{s}}{\rm{e}}{\rm{c}}\,6{3}^{\circ }{\rm{C}}\\  & 1\,{\rm{m}}{\rm{i}}{\rm{n}}\,7{2}^{\circ }{\rm{C}}\end{array}$$

**Table 2:** Primer sequences used in ATAC-Seq.

Primer Name	Sequence	Concentration	Purification
Primer 1 (Ad1)	AATGATACGGCGACCACCGAGATCTACACTCGTCGGCAGCGTCAGATGTG	25 nm	STD
Primer2.1_TAAGGCGA	CAAGCAGAAGACGGCATACGAGATTCGCCTTAGTCTCGTGGGCTCGGAGATGT	25 nm	STD
Primer2.2_CGTACTAG	CAAGCAGAAGACGGCATACGAGATCTAGTACGGTCTCGTGGGCTCGGAGATGT	25 nm	STD
Primer2.3_AGGCAGAA	CAAGCAGAAGACGGCATACGAGATTTCTGCCTGTCTCGTGGGCTCGGAGATGT	25 nm	STD
Primer2.4_TCCTGAGC	CAAGCAGAAGACGGCATACGAGATGCTCAGGAGTCTCGTGGGCTCGGAGATGT	25 nm	STD
Primer2.5_GGACTCCT	CAAGCAGAAGACGGCATACGAGATAGGAGTCCGTCTCGTGGGCTCGGAGATGT	25 nm	STD
Primer2.6_TAGGCATG	CAAGCAGAAGACGGCATACGAGATCATGCCTAGTCTCGTGGGCTCGGAGATGT	25 nm	STD
Primer2.7_CTCTCTAC	CAAGCAGAAGACGGCATACGAGATGTAGAGAGGTCTCGTGGGCTCGGAGATGT	25 nm	STD
Primer2.8_CAGAGAGG	CAAGCAGAAGACGGCATACGAGATCCTCTCTGGTCTCGTGGGCTCGGAGATGT	25 nm	STD
Primer2.9_GCTACGCT	CAAGCAGAAGACGGCATACGAGATAGCGTAGCGTCTCGTGGGCTCGGAGATGT	25 nm	STD
Primer2.10_CGAGGCTG	CAAGCAGAAGACGGCATACGAGATCAGCCTCGGTCTCGTGGGCTCGGAGATGT	25 nm	STD
Primer2.11_AAGAGGCA	CAAGCAGAAGACGGCATACGAGATTGCCTCTTGTCTCGTGGGCTCGGAGATGT	25 nm	STD
Primer2.12_GTAGAGGA	CAAGCAGAAGACGGCATACGAGATTCCTCTACGTCTCGTGGGCTCGGAGATGT	25 nm	STD
Primer2.13_GTCGTGAT	CAAGCAGAAGACGGCATACGAGATATCACGACGTCTCGTGGGCTCGGAGATGT	25 nm	STD
Primer2.14_ACCACTGT	CAAGCAGAAGACGGCATACGAGATACAGTGGTGTCTCGTGGGCTCGGAGATGT	25 nm	STD
Primer2.15_TGGATCTG	CAAGCAGAAGACGGCATACGAGATCAGATCCAGTCTCGTGGGCTCGGAGATGT	25 nm	STD
Primer2.16_CCGTTTGT	CAAGCAGAAGACGGCATACGAGATACAAACGGGTCTCGTGGGCTCGGAGATGT	25 nm	STD
Primer2.17_TGCTGGGT	CAAGCAGAAGACGGCATACGAGATACCCAGCAGTCTCGTGGGCTCGGAGATGT	25 nm	STD
Primer2.18_GAGGGGTT	CAAGCAGAAGACGGCATACGAGATAACCCCTCGTCTCGTGGGCTCGGAGATGT	25 nm	STD
Primer2.19_AGGTTGGG	CAAGCAGAAGACGGCATACGAGATCCCAACCTGTCTCGTGGGCTCGGAGATGT	25 nm	STD
Primer2.20_GTGTGGTG	CAAGCAGAAGACGGCATACGAGATCACCACACGTCTCGTGGGCTCGGAGATGT	25 nm	STD
Primer2.21_TGGGTTTC	CAAGCAGAAGACGGCATACGAGATGAAACCCAGTCTCGTGGGCTCGGAGATGT	25 nm	STD
Primer2.22_TGGTCACA	CAAGCAGAAGACGGCATACGAGATTGTGACCAGTCTCGTGGGCTCGGAGATGT	25 nm	STD
Primer2.23_TTGACCCT	CAAGCAGAAGACGGCATACGAGATAGGGTCAAGTCTCGTGGGCTCGGAGATGT	25 nm	STD
Primer2.24_CCACTCCT	CAAGCAGAAGACGGCATACGAGATAGGAGTGGGTCTCGTGGGCTCGGAGATGT	25 nm	STD

ATAC-Seq primer sequences.

Primer sequences used in ATAC-Seq to amplify transposed DNA.

The five minute extension in the first cycle is critical to allow extension on both ends of the primer after transposition, thereby generating amplifiable fragments. This ensures that downstream quantitative PCR (qPCR) quantitation will not change the complexity of the original library.

#### qPCR

To reduce the GC and size bias in PCR, the appropriate number of PCR cycles (N) was determined using qPCR, allowing us to stop prior to saturation. The samples were kept in the thermocycler following the PCR Amplification reaction, and the qPCR side reaction was run. In a 0.2 mL PCR tube the following were added: 5 μL of DNA PCR amplified DNA, 2 μL of nuclease free H_2_O, 1 μL of 6.25 mM Custom Nextera PCR Primer (Ad1), 1 μL of 6.25 mM Custom Nextera PCR Primer 2 (Ad2.X), 1 μL 9X SYBR Green I, and 5 μL NEBNext High-Fidelity 2X PCR Master Mix. This sample was run in the qPCR instrument with the following cycles:

$$\begin{array}{rl}1\,{\rm{c}}{\rm{y}}{\rm{c}}{\rm{l}}{\rm{e}}: & 30\,{\rm{s}}{\rm{e}}{\rm{c}}\,9{8}^{\circ }{\rm{C}}\\ 20\,{\rm{c}}{\rm{y}}{\rm{c}}{\rm{l}}{\rm{e}}{\rm{s}}: & 10\,{\rm{s}}{\rm{e}}{\rm{c}}\,9{8}^{\circ }{\rm{C}}\\  & 30\,{\rm{s}}{\rm{e}}{\rm{c}}\,6{3}^{\circ }{\rm{C}}\\  & 1\,{\rm{m}}{\rm{i}}{\rm{n}}\,7{2}^{\circ }{\rm{C}}\end{array}$$

To calculate the additional number of cycles needed, a linear plot of Rn versus cycle was generated. This determined the cycle number (N) that corresponds to one-third of the maximum fluorescent intensity.

The remaining 45 mL PCR reaction was run to the cycle number (N) determined by qPCR. Cycles are as follows:

$$\begin{array}{rl}1\,{\rm{c}}{\rm{y}}{\rm{c}}{\rm{l}}{\rm{e}}: & 30\,{\rm{s}}{\rm{e}}{\rm{c}}\,9{8}^{\circ }{\rm{C}}\\ {\rm{N}}\,{\rm{c}}{\rm{y}}{\rm{c}}{\rm{l}}{\rm{e}}{\rm{s}}: & 10\,{\rm{s}}{\rm{e}}{\rm{c}}\,9{8}^{\circ }{\rm{C}}\\  & 1\,{\rm{m}}{\rm{i}}{\rm{n}}\,7{2}^{\circ }{\rm{C}}\\  & 30\,{\rm{s}}{\rm{e}}{\rm{c}}\,6{3}^{\circ }{\rm{C}}\end{array}$$

The amplified library was purified using Qiagen MinElute PCR Purification Kit after the additional PCR. The purified library was eluted in 20 μL of elution buffer (Buffer EB from the MinElute Kit consisting of 10 mM Tris-Cl (pH 8)). It is important to make sure that the column is dry prior to adding elution buffer to avoid ethanol contamination of final library. The amplified library was purified using AMPure XP beads at a 1.8x ratio to get rid of adapter dimers, using 80% ethanol for the wash steps. Sample was eluted in 50 μL of nuclease free H_2_O. The concentration of the DNA eluted from the column should be about 30 nM.

#### Library preparation

The quality of the purified libraries was assessed using a Bioanalyzer High-Sensitivity DNA Analysis kit (Agilent). If libraries contained predominant peaks around 1000 bp, SPRI beads were used to remove these fragments. This was accomplished by first, with a new vial of SPRI beads, performing size selection with various ratios to ensure larger peaks are removed. For example, ratios could include 0.4X, 0.45X, 0.5X. Choose the ratio that removes 1000 bp fragments, but leaves 800 bp fragments. Libraries were eluted in 20 μL of nuclease-free water, and sequenced as described below.

#### Sequencing for ATAC-Seq by Beijing Genomics International (BGI)

Sequencing was conducted by Beijing Genomics International at the Children’s Hospital of Philadelphia. Samples were quality control tested using an Agilent High Sensitivity Screen Tape to confirm average fragment sizes were ~180, 380, 580, 780, and 980 bp. The concentration of each library was measured using a High Sensitivity Qubit Quantification kit, to ensure they were 5.5 nM. Samples were normalized and libraries were pooled together in equal amounts. Samples were then sequenced as paired-ends, 100 bp to an average depth of 80 M reads on a HiSeq. 2500.

#### ATAC-Seq NB-69 and NGP cell lines via Active Motif

Cells were grown as described in Cell Growth and Expansion section of protocol to 70–80% confluence in a 75 mm^2^ tissue culture flasks in 10 mL of media. Following detachment and pelleting, cells were resuspended in 1.0 mL of the appropriate growth media. Cells were triturated into a homogenous single-cell suspension. Using an automated cell counter, the volume for 100,000 cells was determined, and aliquoted into a sterile 1.5 mL eppendorf tube containing 500 μL of sterile 1X PBS. Cells were then centrifuged at 500 × *g* for 5 minutes at 4 °C. The supernatant was carefully aspirated off, and the cells were resuspended in 500 μL of growth media with 5% DMSO. The sample was transferred to a 1.7 mL microfuge tube on ice. Cells were frozen with a slow cooling to minimize cell lysis. Samples were shipped on dry ice to Active Motif (1914 Palomar Oaks Way, Ste 150, Carlsbad, CA 92008) following the instructions listed at on the Sample Submission Form, downloaded from www.activemotif.com/sample-submission. Samples were prepared and sequenced following Active Motif’s ATAC-Seq proprietary protocol. Cells were thawed in a 37 °C water bath, pelleted, washed with cold PBS, and tagmented as previously described^18^, with some modifications based on^19^. Cell pellets were resuspended in lysis buffer, pelleted, and tagmented using the enzyme buffer provided in the Nextera Library Prep Kit (Illumina). Tagmented DNA was then purified using the MinElute PCR purification kit (Qiagen), amplified with 10 cycles of PCR, and purified using Agencourt AMPure SPRI beads (Beckman Coulter). The resulting material was quantified using the KAPA Library Quantification Kit for Illumina platforms (KAPA Biosystems) and sequenced with PE42 sequencing on the NextSeq. 500 sequencer (Illumina).

### ChIP-Seq data analysis

FASTQ quality was assessed using FastQC v0.11.4 (http://www.bioinformatics.babraham.ac.uk/projects/fastqc/) and sequences were adapter- and quality-trimmed using default parameters for Trim Galore v.0.4.0 and CutAdapt v.1.12^20,21^. MultiQC v1.4 was used to aggregate FastQC results across all samples, with the report available on Figshare^22^. Since multiple sequencers were used, FASTQ phred sequencing scores^23^ were calculated using a perl script (https://raw.githubusercontent.com/douglasgscofield/bioinfo/master/scripts/phredDetector.pl). This value was used as input into the alignment algorithm. The bwa v.0.7.12 samse^24^ was used to align the reads to hg19 reference genome and Picard tools v.2.17.9-SNAPSHOT^25^ was used to remove duplicates. Fragment sizes were estimated using MaSC 1.2.1^26^ and these values were used as input into the –extsize argument of MACS2 v.2.1.1^27^ for narrow peak calling (transcription factors) or broad peak calling (histone marks). Broad peaks were called significant using a q-value (minimum False Discovery Rate) cut off of 0.10 and narrow peaks at a q-value cutoff of 0.05. Results were returned in units of signal per million reads to get normalized peak values. Repetitive centromeric, telomeric and satellite regions known to have low sequencing confidence were removed using blacklisted regions defined by the ENCODE project: http://mitra.stanford.edu/kundaje/akundaje/release/blacklists/hg19-human/wgEncodeHg19ConsensusSignalArtifactRegions.bed.gz. The resulting filtered peakfiles were used as input into Homer v4.10.4 for gene annotation and motif analysis.

### ATAC-Seq data analysis

Samples were quality-controlled and trimmed as described in Chip-Seq Analysis. FASTQ files were aligned using bwa aln for BGI samples (100 bp reads) and bwa mem for Active Motif samples (42 bp reads). Reads with mapping quality <10 were discarded. Biological duplicate BAMs were merged using Picard v.2.17.9-SNAPSHOT. Broad peaks were called using –extsize 200, –shift 100, –nomodel. Results were returned in units of signal per million reads to get normalized peak values. Finally, repetitive centromeric, telomeric and satellite regions known to have low sequencing confidence were removed using merged blacklisted regions defined by the ENCODE project: http://mitra.stanford.edu/kundaje/akundaje/release/blacklists/hg19-human/wgEncodeHg19ConsensusSignalArtifactRegions.bed.gz.

### ChIP-Seq quality control metrics

We investigated three metrics to assess ChIP-seq quality. To calculate enrichment of reads within peaks we determined the FRiP score using deeptools2^28^. The FRiP score is defined as the fraction of reads that fall within a peak divided by the total number of reads. To measure read enrichment independent of peak calling we calculated the NSC (normalized strand cross-correlation) and the RSC (relative strand cross-correlation) using phantompeakqualtools^29,30^ as part of the ENCODE ChIP-seq processing pipeline. All ChIP-Seq data passed quality control and results are reported in Online Table 3.

### ATAC-Seq quality control metrics

To compare reproducibility between ATAC-seq biological replicates we performed irreproducible discovery rate (IDR) analysis using scripts downloaded from https://github.com/nboley/idr. Peaks passing the suggested threshold (IDR < = 0.05%) between two replicates were kept. The ratio between the number of peaks between true replicates (Nt) and pooled pseudoreplicates (Np) was calculated. In accordance with ENCODE guidelines, we confirmed that at least 50% of true replicate IDR analysis based peaks (Nt) were identified in the IDR comparison of pseuduoreplicates (Np): Np/Nt < 2. A similar analysis was done with self-pseudoreplicates (N1 and N2). We confirmed that the ratio between Np/Nt or N1/N2 was <2. All ATAC-seq data passed IDR results and are reported in Online Table 4. Peakfiles resulting from IDR analysis are available from FigShare^31^.

### Super-enhancer calling and comparison

Super-enhancers (SEs) were called from H3K27Ac BAM files using the default parameters of LILY (https://github.com/BoevaLab/LILY), which includes correction for copy number variation inherently present in cancer samples. Enhancers were classified into SEs, enhancers, and promoters and annotated using Homer v4.10.4. Scripts to run LILY can be found on Github (https://github.com/marislab/epigenomics-data-descriptor). SEs were also called from H3K27Ac MACS2 peaks using ROSE v.0.1 (https://bitbucket.org/young_computation/rose/src/master/) using default parameters and annotated using Homer v4.10.4. SEs which overlapped with the MYCN locus (hg19, chr2:16080683-16087129) were removed from the analysis. SE genes which we annotated as transcription factors^32^ were used for comparison to two literature studies^33,34^.

### Heatmap preparation

The 5,000 most significant (sorted by highest -log_10_(p-value) and -log_10_(q-value)) MYCN peaks for each of the five MYCN amplified cell line were intersected using bedtools. Heatmaps were generated for regions +/−4 kb from the transcription start site (TSS) for the 5,046 peaks common to at least four MYCN amplified cell lines. Heatmaps were created for LA-N-5 and NB-69 at loci annotated as enhancers, SEs, and promoters-TSS by LILY. All ChIP-seq heatmaps were created using deepTools 3.2.0 package plotHeatmap tool^28^. The code and parameters used to generate heatmaps can be found on GitHub (https://github.com/marislab/epigenomics-data-descriptor).

### Cell line authentication

All cell lines were STR-authenticated by Guardian Forensic Sciences (Abington, PA) using the GenePrint 24 (Promega, #B1870).

## Data Records

Raw, concatenated FASTQ files were deposited in Sequence Read Archive under the SRA study accessions SRP223941^35^, SRP223977^36^, and SRP223942^37^. Processed BIGWIG files for all sequencing data were deposited into the Gene Expression Omnibus (GEO) under SuperSeries Accession Number GSE138315^38^. MYCN and MYC ChIP-Seq data for the Kelly and NGP cell lines were deposited into GEO under Accession Number GSE94782^39^, all other MYCN and MYC ChIP-Seq were deposited under Accession Number GSE138295^40^, histone ChIP-Seq data were deposited under Accession Number GSE138314^41^, and ATAC-Seq data were deposited under Accession Number GSE138293^42^. Homer motif analysis and motif files are available on FigShare^22,31,43^.

## Technical Validation

**Fig. 2:**
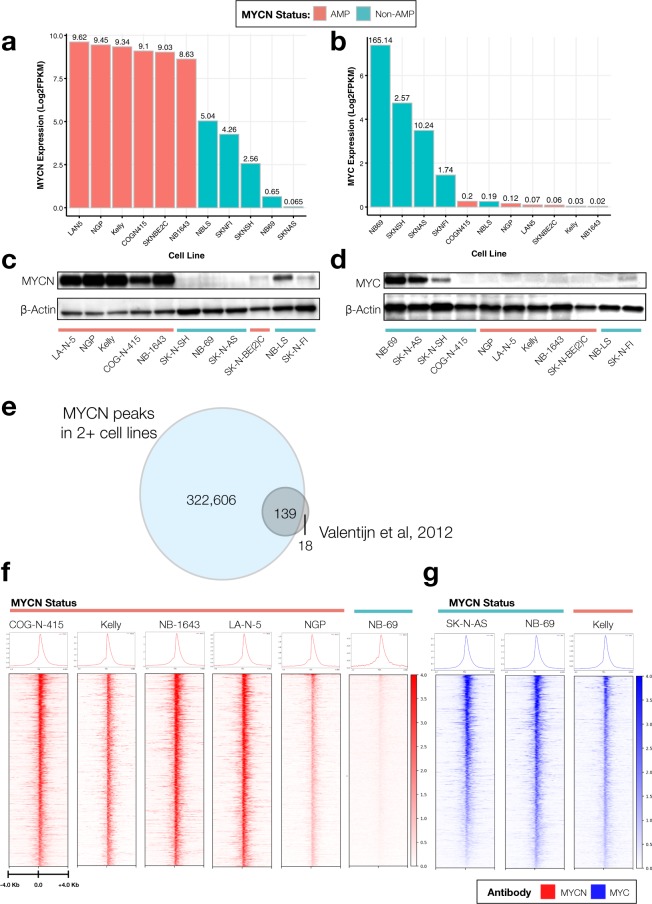
Comparison of MYCN and MYC binding based on MYCN amplification status. The log_2_ FPKM mRNA expression of *MYCN* (**a**) and *MYC* (**b**) in all neuroblastoma cell lines assayed herein. Protein expression for MYCN (**C**) and MYC (**d**) in a subset of cell lines. (**e**) Comparison of unique MYCN peaks called in at least two of the MYCN ChIP-Seq samples (blue) to known MYCN regulated genes (gray)^45^ demonstrates concordance of MYCN ChIP-Seq to MYCN ChIP-ChIP. (**f**) The top 1,335 MYCN peaks (p < 0.05, q < 0.05) are plotted as ChIP-Seq heatmaps for five MYCN amplified cell lines (COG-N-415, Kelly, NB-1643, LAN-5, and NGP) and one MYCN non-amplified cell line (NB-69). All heat map densities ranges from +/−4.0 Kb from the TSS, with average signal plots shown above. (**g**) MYC ChIP-Seq heat maps for the same peaks in two MYCN non-amplified lines (SK-N-AS, and NB-69) and one MYCN amplified cell line (Kelly) showing redundant binding of MYC in non-amplified cell lines.

Prior to selecting cell lines for MYCN and MYC profiling, we assessed RNA expression (Fig. 2a,b) and protein expression (Fig. 2c,d) across a subset of neuroblastoma cell lines. NB-LS, while *MYCN* non-amplified, has substantial MYCN RNA and protein expression^44^, but was not chosen, as we restricted MYCN ChIP-Seq to *MYCN* amplified cell lines plus one negative control. SK-N-BE(2)-C, a *MYCN* amplified cell line, showed high *MYCN* mRNA expression, but surprisingly low protein expression, and thus was excluded. The remaining cell lines had concordant *MYCN* and *MYC* mRNA and protein expression, thus, COG-N-415, KELLY, NB-1643, LA-N-5, and NGP were chosen for MYCN ChIP-Seq while NB-69, SK-N-AS, and SK-N-SH were chosen for MYC ChIP-Seq. As additional controls, we performed MYCN ChIP-Seq in the *MYCN* non-amplified line NB-69, and MYC ChIP-Seq on the *MYCN* amplified cell line KELLY. To validate the MYCN and MYC ChIP-Seq antibodies, we first intersected loci bound by MYCN in two or more cell lines and of the 157 MYCN transcriptional targets previously reported using ChIP-on-ChIP^45^, found 139 loci occupied by the MYCN via ChIP-Seq (Fig. 2e). Next, we integrated the top 5,000 MYCN peaks from each *MYCN* amplified cell line. We generated heatmaps for the peaks (1,335) which overlapped in all five cell lines (as defined in ***Heatmap Preparation***) and depict occupancy of MYCN (Fig. 2f) and MYC (Fig. 2g) at these sites. As expected, the MYCN amplified cell lines COG-N-415, KELLY, NB-1643, LA-N-5, and NGP show similar binding profiles, while the negative control MYCN non-amplified line NB-69 depicted an absence of binding for MYCN at the same loci. Importantly, Homer motif analysis of the 34,906 target sequences bound by MYC in NB-69 were significantly enriched (Benjamini q-value < 0.001) for the canonical CACGTG e-box motif, while this motif was absent from the 112 target sequences found in the NB-69 MYCN ChIP-Seq sample. We observed MYC bound to the same loci in the MYCN non-amplified cell lines, SK-N-AS, SK-N-SH, and NB-69 as well as the *MYCN* amplified and low MYC-expressing line KELLY (Fig. 2g), and observed shared CACGTG motif binding for both MYCN and MYC in KELLY, supporting the notion of redundant functionality of MYC family protein members. To further validate both the specificity and functional redundancy of the MYCN and MYC ChIP-Seq, we assessed MYCN and MYC binding to transcriptional targets of an 18-gene MYC family (MYCN/MYC/MYCL1) activity signature^46^ in KELLY (MYCN and MYC) and SKNBE(2)C (MYCN) cell lines alongside six non-MYC family core regulatory TFs (*ASCL1, GATA3, HAND2, ISL1, PHOX2B, TBX2*) from publicly-available ChIP-Seq data (*ASCL1:* GEO accession number GSE120074 and *GATA3, HAND2, ISL1, PHOX2B, TBX2:* GEO accession number GSE94824) reprocessed with our pipeline (see Methods). Supplemental Fig. 2 shows the binding patterns for four of the 18 genes: *APEX1, NME1, ENO1*, and *ODC1*. *APEX1, NME1*, and *ENO1* are not bound by the six non-MYC family core regulatory TFs (*ASCL1, GATA3, HAND2, ISL1, PHOX2B, TBX2*), while *ASCL1* shows binding at *ODC1* because it recognizes the e-box motif, CANNTG. Altogether, these data demonstrate specificity of MYCN and MYC antibodies and functional redundancy of MYCN and MYC proteins.

**Fig. 3:**
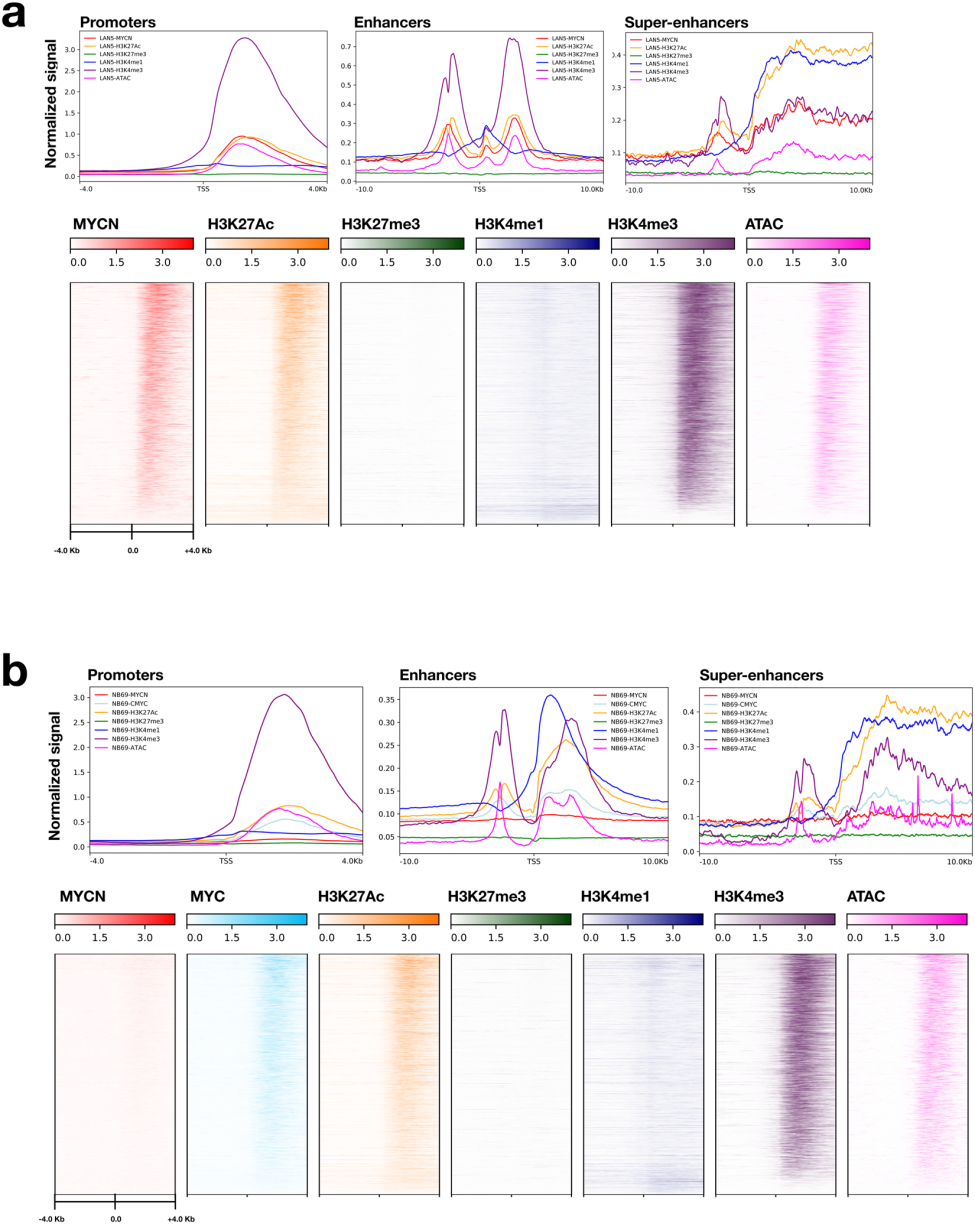
Validation of ChIP-Seq promoter, enhancer, and open chromatin occupancy. Binding densities of MYCN, MYC, histone antibodies, and open chromatin for promoter regions of the MYCN-amplified cell line LA-N-5 (**a**) and the MYCN non-amplified cell line NB-69 (**b**) are depicted (+/−4.0 Kb from gene TSS) and distinct profiles are shown for promoters (+/−4.0 Kb from gene TSS), enhancers (+/−10.0 Kb from gene TSS), and SEs (+/−10.0 Kb from gene TSS). For LA-N-5: N_promoters-TSS_ = 4,662, N_enhancers_ = 25,601, N_SE_ = 826, and for NB-69: N_promoters-TSS_ = 4,718, N_enhancers_ = 31,769, N_SE_ = 667.

Next, we evaluated genome-wide binding densities of the histone antibodies and assessed open chromatin by plotting binding of one *MYCN* amplified cell line LA-N-5 (Fig. 3a), and one *MYCN* non-amplified cell line NB-69 (Fig. 3b). Of note, cell-line specific promoters are located in regions of open chromatin and strongly occupied by narrow regions of H3K4me3 and devoid of H3K27me3 and H3K4me1, as expected. The majority of promoters are also occupied by MYCN in LA-N-5 and MYC in NB-69. Enhancers have bivalent marking of MYCN, H3K4me3, H3K27Ac, open chromatin, and absence of H3K27me3. SEs are broadly marked by MYCN, H3K4me3, H3K27Ac, H3K4me1, and open chromatin.

**Fig. 4:**
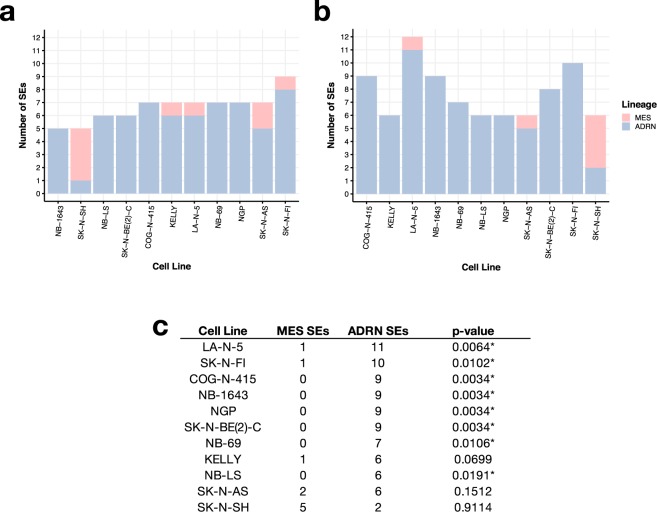
Super-enhancer calls correctly determine neuroblastoma cell line lineage (**a**) Total number of lineage-specific SEs called from ROSE and (**b**) LILY per cell line (ADRN = adrenergic, MES = mesenchymal subtype). (**c**) Total number of lineage-specific SEs called when combining ROSE and LILY results. One-tailed fisher’s exact test p-values are listed (*denotes significance at an α level of 0.05).

Finally, we used our H3K27Ac ChIP-Seq data to compare SE prediction of cell line lineage in our dataset compared to those reported in two other publications describing the SE landscape in neuroblastoma (Fig. 4 and Supplemental Fig. 1). Boeva and colleagues identified 4,791 SE-associated genes in Table S3 to identify core regulatory transcriptional circuitry in neuroblastoma using 25 cell lines^33^. Four cell lines were common to our study: SK-N-BE(2)C, SK-N-FI, SK-N-AS, and NB-69. Therefore, to validate our H3K27Ac ChIP-Seq, we utilized the same algorithm (LILY, see Methods) to call SEs from our H3K27Ac data, and restricted comparison analyses to genes defined as transcription factors (TFs), as defined by core regulatory circuitry^32^. We annotated 396 of the SEs reported by Boeva and colleagues as transcription factors and found 59–85% concordance of our TF SE calls (Supplemental Fig. 1). While a majority of SEs called in each of our cell lines was concordant with Boeva and colleagues, the high variance in total number of SEs called likely stems from the diversity of cell lines in both studies, as well as pipeline processing and filtering parameters. We were unable to directly compare methods without their code and raw data readily available. Thus, we additionally compared our TF SE calls to those from an independent neuroblastoma study^34^ which used the ROSE algorithm (see Methods) and reported smaller SE genesets (Online Table 5) driving the lineage-specific mesenchymal (MES, N = 20 TFs) and adrenergic (ARDN, N = 18 TFs) subtypes. To mimic the analysis performed by van Gronigen and colleagues, we ran ROSE on our H3K27Ac ChIP-Seq data and removed any peaks which overlapped the *MYCN* locus (see Methods) to account for false SE calls due to *MYCN* amplification. There were no common neuroblastoma cell lines between van Gronigen and colleagues study and the lines used in our study. We assessed the number of MES or ADRN SE-associated TFs detected in each of our study and found between five and eight ADRN SEs were detected using ROSE (Fig. 4a) and between five and 11 ADRN SEs were detected using LILY (Fig. 4b). SK-N-SH has a known MES subtype; its subclone, SH-SY-5Y, was profiled as MES by van Gronigen and colleagues. Combining the calls, we were able to significantly (Fisher’s exact test, p < 0.05) validate ADRN subtypes in eight of the ten cell lines we profiled (Fig. 4c). Interestingly, SK-N-AS contains SEs from both subtypes and thus may reflect a heterogeneous cell line. Specific SEs are reported per algorithm per cell line in Online Table 5. As further validation, we re-analyzed publicly-available SK-N-SH H3K27Ac (Biosample SAMN05733860, Run SRR5338927) and SK-N-SH Input (Biosample SAMN05733844, Run SRR5471111) ChIP-Seq data (GEO accession GSM2534162) using the same peak-calling and SE pipelines used on our data (see Methods). We observed enhancer binding (H3K27Ac) and open chromatin (ATAC) at the same loci we observe strong MYC occupancy (Supplemental Fig. 1A). Further, we assessed concordance of SEs called in SK-N-SH with those previously reported and found 76% of TF SEs called in SK-N-SH in common with those from Boeva, et. al, similar to our findings.

Together, we have validated both MYCN and MYC ChIP-Seq antibodies for use in ChIP-Seq, as well as genome-wide occupancy profiles for histone markers and open chromatin across a cohort of neuroblastoma cell lines. We ran two algorithms (LILY and ROSE) and compared our data to two independent datasets to validate reproducibility of lineage-specific SEs in neuroblastoma cell lines. Finally, we demonstrate integration of publicly-available H3K27Ac data from SK-N-SH with our MYC ChIP-Seq and ATAC-Seq data, and show reproducibility of SE calls between the publicly-available data and two independent reports. These data should be a valuable resource to the childhood cancer and MYC research communities.

## Usage Notes

Here, we provide raw FASTQ and bigwigs for a comprehensive, validated ChIP-Seq (MYCN, MYC, H3K27Ac, H3K27me3, H3K4me3, and H3K4me1) and ATAC-Seq neuroblastoma cell line dataset which can be coupled with our previous RNA-Seq profiling dataset^16^ to interrogate novel transcriptional regulation in this disease. For example, the H3K27me3 ChIP-Seq can be used to identify genes being repressed via the PRC2 complex, while H3K27Ac and H3K4me1 ChIP-Seq can be used to interrogate promoter-enhancer mechanisms. CSI-ANN can be used to integrate histone ChIP-Seq data to predict regulatory DNA segments^47^, and IM-PET can use the results from CSI-ANN to predict enhancer-promoter interactions without the need for Hi-C data^13^. Additionally, chromatin states can be inferred^48,49^, and these data can be later integrated with whole exome or genome sequencing data or genome-wide association studies to identify molecular alterations driving transcriptional regulatory marked by histone marks or open chromatin.

All data are openly-available from GEO as described in the Data Records section.

### Supplementary information

Supplemental Figures 1 and 2

## Data Availability

Code for IDR analysis, SE calling, filtering, heatmap generation is available on Github^50^.

## Online-only Tables

**Online Table 1:** Neuroblastoma cell lines profiled in this study.

		Histone ChIP-Seq																Transcription Factor								Other			
Cell Line	MYCN Status	H3K27Ac				H3K27me3				H3K4me1				H3K4me3				MYCN				MYC				ATAC			
		Sequencing Facility	Sequencer	Read Length	PE or SE	Sequencing Facility	Sequencer	Read Length	PE or SE	Sequencing Facility	Sequencer	Read Length	PE or SE	Sequencing Facility	Sequencer	Read Length	PE or SE	Sequencing Facility	Sequencer	Read Length	PE or SE	Sequencing Facility	Sequencer	Read Length	PE or SE	Sequencing Facility	Sequencer	Read Length	PE or SE
COG-N-415	amp	Jefferson	NextSeq. 500	75 bp	SE	Jefferson	NextSeq. 500	75 bp	SE	Jefferson	NextSeq. 500	75 bp	SE	Jefferson	NextSeq. 500	75 bp	SE	Jefferson	NextSeq. 500	75 bp	SE	NA	NA	NA	NA	CHOP	HiSeq. 2500	100 bp	PE
COG-N-440	amp	Jefferson	NextSeq. 500	75 bp	SE	Jefferson	NextSeq. 500	75 bp	SE	Jefferson	NextSeq. 500	75 bp	SE	Jefferson	NextSeq. 500	75 bp	SE	NA	NA	NA	NA	NA	NA	NA	NA	CHOP	HiSeq. 2500	100 bp	PE
COG-N-453	non-amp	Jefferson	NextSeq. 500	75 bp	SE	Jefferson	NextSeq. 500	75 bp	SE	Jefferson	NextSeq. 500	75 bp	SE	Jefferson	NextSeq. 500	75 bp	SE	NA	NA	NA	NA	NA	NA	NA	NA	CHOP	HiSeq. 2500	100 bp	PE
KELLY	amp	Jefferson	NextSeq. 500	75 bp	SE	Jefferson	NextSeq. 500	75 bp	SE	Jefferson	NextSeq. 500	75 bp	SE	Jefferson	NextSeq. 500	75 bp	SE	CHOP	MiSeq	50 bp	SE	Jefferson	NextSeq. 500	75 bp	SE	CHOP	HiSeq. 2500	100 bp	PE
LA-N-5	amp	Jefferson	NextSeq. 500	75 bp	SE	Jefferson	NextSeq. 500	75 bp	SE	Jefferson	NextSeq. 500	75 bp	SE	Jefferson	NextSeq. 500	75 bp	SE	Jefferson	NextSeq. 500	75 bp	SE	NA	NA	NA	NA	CHOP	HiSeq. 2500	100 bp	PE
NB-1	amp	Jefferson	NextSeq. 500	75 bp	SE	Jefferson	NextSeq. 500	75 bp	SE	Jefferson	NextSeq. 500	75 bp	SE	Jefferson	NextSeq. 500	75 bp	SE	NA	NA	NA	NA	NA	NA	NA	NA	CHOP	HiSeq. 2500	100 bp	PE
NB-1643	amp	Jefferson	NextSeq. 500	75 bp	SE	Jefferson	NextSeq. 500	75 bp	SE	Jefferson	NextSeq. 500	75 bp	SE	Jefferson	NextSeq. 500	75 bp	SE	Jefferson	NextSeq. 500	75 bp	SE	NA	NA	NA	NA	CHOP	HiSeq. 2500	100 bp	PE
NB-69	non-amp	Jefferson	NextSeq. 500	75 bp	SE	Jefferson	NextSeq. 500	75 bp	SE	Jefferson	NextSeq. 500	75 bp	SE	Jefferson	NextSeq. 500	75 bp	SE	Jefferson	NextSeq. 500	75 bp	SE	Jefferson	NextSeq. 500	75 bp	SE	Active Motif	NextSeq. 500	42 bp	PE
NB-LS	non-amp	Jefferson	NextSeq. 500	75 bp	SE	Jefferson	NextSeq. 500	75 bp	SE	Jefferson	NextSeq. 500	75 bp	SE	Jefferson	NextSeq. 500	75 bp	SE	NA	NA	NA	NA	NA	NA	NA	NA	CHOP	HiSeq. 2500	100 bp	PE
NGP	amp	Jefferson	NextSeq. 500	75 bp	SE	Jefferson	NextSeq. 500	75 bp	SE	Jefferson	NextSeq. 500	75 bp	SE	Jefferson	NextSeq. 500	75 bp	SE	Jefferson	NextSeq. 500	75 bp	SE	NA	NA	NA	NA	Active Motif	NextSeq. 500	42 bp	PE
SK-N-AS	non-amp	Jefferson	NextSeq. 500	75 bp	SE	Jefferson	NextSeq. 500	75 bp	SE	Jefferson	NextSeq. 500	75 bp	SE	Jefferson	NextSeq. 500	75 bp	SE	NA	NA	NA	NA	Jefferson	NextSeq. 500	75 bp	SE	CHOP	HiSeq. 2500	100 bp	PE
SK-N-BE(2)-C	amp	Jefferson	NextSeq. 500	75 bp	SE	Jefferson	NextSeq. 500	75 bp	SE	Jefferson	NextSeq. 500	75 bp	SE	Jefferson	NextSeq. 500	75 bp	SE	Jefferson	NextSeq. 500	75 bp	SE	NA	NA	NA	NA	CHOP	HiSeq. 2500	100 bp	PE
SK-N-FI	non-amp	Jefferson	NextSeq. 500	75 bp	SE	Jefferson	NextSeq. 500	75 bp	SE	Jefferson	NextSeq. 500	75 bp	SE	Jefferson	NextSeq. 500	75 bp	SE	NA	NA	NA	NA	NA	NA	NA	NA	CHOP	HiSeq. 2500	100 bp	PE
SK-N-SH	non-amp	*	*	*	*	*	*	*	*	*	*	*	*	*	*	*	*	*	*	*	*	*	*	*	*	*	*	*	*

*External data available from ENCODE.

Identification of which cell lines were used to perform ChIP-Seq and ATAC-Seq, along with their MYCN amplification status.

**Online Table 2:** Materials and reagents used in this study.

Protocol Section		Reagents	Manufacture	Identifier	Volume/Concentration
Cell Growth and Expansion		RPMI 1640 (with 25 mM HEPES)	Thermo Fisher Scientific	22400089	
		Iscove’s IMDM	Thermo Fisher Scientific	12440053	
		L-glutamine	Thermo Fisher Scientific	25030081	2 mM
		Hyclone Fetal Bovine Serum (FBS)	Fisher Scientific	SH3007.13	10% (RMPI)
					20% (IMDM)
		Antibiotic/antimycotic	Thermo Fisher Scientific	15240062	1.0%
		Insulin/Transferrin/Selenium (ITS)	Corning Life Science	354351	0.1% (IMDM)
Immunobltoing	Cell Lysis Buffer (1 mL)	Cell Lysis Buffer	Cell Signaling	9803	100 μL
		PMSF	Cell Signaling	8553S	20 μl (100 mM)
		Phosphatase Inhibitor Cocktail 2	Sigma Aldrich	P5726	10 μL
		Phosphatase Inhibitor Cocktail 3	Sigma Aldrich	P0044	10 μL
		PBS	Gibco	14190-136	860 μL
		4X Laemmli Sample Buffer	BioRad	1610747	
		Pierce BCA Protein Assay Kit	Thermo Scientific	23225	
		4–15% Criterion TGX Precast Midi Protein Gel	BioRad	5671085	
		N-MYC	Cell Signaling	9405 S	1:1000
		MYC	Cell Signaling	5605	1:800
		B-Actin	Cell Signaling	4976 S	1:5000
		Rabbit Secondary	Millipore	AP132P	1:2000-1:5000
ChIP-Seq	Formaldehyde Solution	37% Formaldehyde	Sigma Aldrich	F8775	11%
		5.0 M NaCl	Sigma Aldrich	S5150	0.1 M
		0.5 EDTA (pH 8.0)	Invitrogen	15575-038	1 mM
		1.0 M HEPES (pH 7.9)	Gibco	15630-080	50 mM
		Nuclease-free H2O	Ambion	AM9937	
	Glycine Solution	Glycine (mw 75)	Sigma Aldrich	G7403	2.5 M
		Nuclease-free H2O	Ambion	AM9937	
	Other Cell Fixation Reagents:	PMSF	Cell Signaling	8553 S	100 mM
	Antibodies	N-Myc	Active Motif	#61185	6 μL
		c-Myc	Santa Cruz N262	#sc-764	20 μl
		H3K4me1	Active Motif	#39297	5 μl
		H3K4me3	Active Motif	#39159	3 μl
		H3K27Ac	Active Motif	#39133	4 μg
		HeK27me3	Millipore	#07-449	4 μg
		H3K4me1	Active Motif	#39297	5 μl
	FA Lysis Buffer	1.0 M HEPES (pH 7.5)	Gibco	15630-080	50 mM
		NaCl	Sigma	S5150	140 mM
		EDTA (pH 8.0)	Invitrogen	15575-038	1 mM
		Triton-X-100	Sigma	9002-93-1	0.10%
		SDS	Invitrogen	15553-035	0.10%
		Sodium deoxycholate	VWR	97062-028	0.10%
		Protease Inhibitors	Thermo Scientific	88666	
		DTT	Fisher	CAS 3483-12-3	1 mM
	ChIP Wash Buffer	Tris-HCl (pH 8.0)	Invitrogen	15568-025	20 mM
		NaCl	Sigma	S5150	150 mM
		EDTA (pH 8.0)	Invitrogen	15575-038	2 mM
		Triton-X-100	Sigma	9002-93-1	1.00%
		SDS	Invitrogen	15553-035	0.10%
		Sodium deoxycholate	VWR	97062-028	0.10%
	Final Wash Buffer	Tris-HCl (pH 8.0)	Invitrogen	15568-025	20 mM
		NaCl	Sigma	S5150	500 mM
		EDTA (pH 8.0)	Invitrogen	15575-038	2 mM
		Triton-X-100	Sigma	9002-93-1	1.00%
		SDS	Invitrogen	15553-035	0.10%
		Tris-HCl (pH 8.0)	Invitrogen	15568-025	20 mM
	Other ChIP-Seq Reagents	NEB Ultra Kit	New England Biosciences	E7370S	
		Protein A/G Beads	Thermo Scientific	26162	
ATAC-Seq	ATAC-Seq Lysis Buffer	Tris-HCl (pH 7.4)	Invitrogen	15567027	10 mM
		NaCl	Sigma	S5150	10 mM
		MgCl_2_	Fisher	7791-18-6	3 mM
		lgepal CA-630, molecular biology grade	Sigma	I8896	0.10%
	Other ATAC-Seq Reagents	1X Phosphate Buffered Saline (PBS)	Gibco	14190-136	10 mM
		TDB	Illumina	FC-121-1030 (2X reaction buffer from Nextera Kit)	2X
		TDE1	Illumina	FC-121-30 (Nextera Tn5 Transposase from Nextera Kit)	
		Nuclease-free H_2_O	Ambion	AM9937	
		NEBNext High-Fidelity 2X PCR Master Mix	NEB	M0541	
		10,000X SYBR Green	Invitrogen	S-7563	9X
		MinElute PCR Purification Kit	Qiagen	28104	
		AMPure XP Beads	Beckman Coulter	A63880	
		SPRI Beads	Beckman Coulter	B23317	

Information regarding the resources used for obtaining the ChIP-Seq and ATAC-Seq data. This includes manufacture, item number or identification code, and the volume or concentration used.

**Online Table 3:** ChIP-Seq Quality Control Metrics.

ChIP-seq samples	Total number of mapped reads	NSC	RSC	Quality Tag	FRiP
COGN415-H3K27Ac	47176895	1.198422	1.272757	1	0.474845
COGN415-H3K27me3	36536236	1.071534	1.158632	1	0.49757
COGN415-H3K4me1	46916556	1.07441	1.234691	1	0.495989
COGN415-H3K4me3	35108977	1.737172	1.165273	1	0.665681
KELLY-H3K27Ac	40396065	1.149861	1.251872	1	0.535178
KELLY-H3K27me3	40796047	1.061208	0.928446	0	0.527917
KELLY-H3K4me1	40245244	1.098321	1.204422	1	0.6152
KELLY-H3K4me3	39466119	1.591399	1.190163	1	0.654777
LAN5-H3K27Ac	43885147	1.203236	1.20255	1	0.519087
LAN5-H3K27me3	34543494	1.075239	0.97775	0	0.497408
LAN5-H3K4me1	56164251	1.077862	1.15542	1	0.529717
LAN5-H3K4me3	31858899	1.764651	1.176897	1	0.619316
NB1643-H3K27Ac	41742217	1.296535	1.200707	1	0.52952
NB1643-H3K27me3	23005654	1.039613	0.595513	0	0.360867
NB1643-H3K4me1	50897016	1.122099	1.183034	1	0.567832
NB1643-H3K4me3	37634501	1.532691	1.205901	1	0.568343
NB69-H3K27Ac	53900803	1.167153	1.186056	1	0.38709
NB69-H3K27me3	34409193	1.107075	0.980233	0	0.322941
NB69-H3K4me1	42335848	1.123224	1.159933	1	0.568245
NB69-H3K4me3	27850317	2.250138	1.150775	1	0.707439
NBLS-H3K27Ac	50890146	1.179172	1.215066	1	0.505567
NBLS-H3K27me3	35015208	1.09364	1.030182	1	0.489031
NBLS-H3K4me1	46236749	1.10292	1.169135	1	0.570663
NBLS-H3K4me3	50235082	1.553831	1.101005	1	0.559603
NGP-H3K27Ac	38313036	1.361727	1.198959	1	0.568805
NGP-H3K27me3	37472545	1.077646	1.057799	1	0.536769
NGP-H3K4me1	48180227	1.103056	1.186526	1	0.565611
NGP-H3K4me3	36776488	1.560065	1.190639	1	0.604321
SKNAS-H3K27Ac	44588659	1.193814	1.185694	1	0.51158
SKNAS-H3K27me3	35294269	1.097313	1.054037	1	0.509914
SKNAS-H3K4me1	44751199	1.080169	1.217051	1	0.529041
SKNAS-H3K4me3	37205051	1.59864	1.152108	1	0.552572
SKNBE2C-H3K27Ac	41843000	1.321413	1.250426	1	0.519484
SKNBE2C-H3K27me3	38128223	1.05271	1.088533	1	0.372794
SKNBE2C-H3K4me1	40497385	1.085463	1.219962	1	0.43465
SKNBE2C-H3K4me3	35566334	1.624385	1.192349	1	0.555207
SKNFI-H3K27Ac	29596790	1.256625	1.24649	1	0.51058
SKNFI-H3K27me3	43286296	1.050568	0.964636	0	0.404905
SKNFI-H3K4me1	47444355	1.087186	1.189153	1	0.431344
SKNFI-H3K4me3	33682220	1.892165	1.176541	1	0.596417
SKNSH-H3K27Ac	1483696	1.784565	1.831717	2	0.298579
COGN415-NMYC-20171205	29461253	1.147548	1.317602	1	0.335752
KELLY-CMYC-20171205	19638904	1.092233	1.286621	1	0.112855
KELLY-MYCN-20150914	22082714	1.504426	1.186748	1	0.439206
LAN5-NMYC-20171205	29364683	1.242474	1.273926	1	0.460691
NB1643-NMYC-20171205	22694640	1.238667	1.329251	1	0.440359
NB69-CMYC-20171205	28528819	1.117398	1.233859	1	0.13737
NB69-NMYC-20171205	18647527	1.049936	0.852352	0	0.007798
SKNAS-CMYC-20171205	27536490	1.179348	1.250418	1	0.183257
SKNSH-CMYC-20171205	8773023	1.331705	1.19801	1	0.157383

Total number of reads, FRiP score, RSC, and NSC per sample.

**Online Table 4:** ATAC-seq IDR Analysis.

Samples	IDR (Nt)	# Total Peaks	IDR (Np)	# Total Pseudo Rep Pool Peaks	Np/Nt (<2)	N1	Rep1_Self_Pseudo Peaks	N2	Rep2_Self_Pseudo Peaks	N1/N2 (<2)
COGN415	27051	55764	31677	66503.000	1.171010314	14704	35064	13931	31330.000	1.055487761
COGN440	7218	22089	8932	26076.000	1.237461901	1906	9986	4824	17022.000	0.395107794
COGN453	26805	49832	31053	56091.000	1.158477896	19455	39812	9630	25545.000	2.020249221
KELLY	10648	25971	12907	28929.000	1.212152517	7209	19757	4689	14466.000	1.537428023
LAN5	30751	60243	46583	79014.000	1.514845046	14936	38632	34164	56074.000	0.437185341
NB1643	25220	49715	27600	55257.000	1.094369548	9974	27819	14955	37296.000	0.666934136
NB1	25180	43124	28711	47781.000	1.140230342	14999	29416	14301	28969.000	1.048807776
NB69	46014	82061	51838	85805.000	1.126570174	32369	58267	43363	86770.000	0.746465881
NBLS	17371	35110	19221	39658.000	1.106499338	10644	27571	4995	17682.000	2.130930931
NGP	44934	73646	45296	74607.000	1.00805626	29873	58165	27529	51986.000	1.085146573
SKNAS	15621	35518	17270	37810.000	1.105563024	8003	24257	6290	21135.000	1.272337043
SKNBE2C	3395	13236	4088	15254.000	1.204123711	1105	5957	1640	6912.000	0.673780488
SKNFI	57416	77782	59856	81540.000	1.042496865	40617	59269	38239	58950.000	1.062187819
SKNSH	32737	52860	36584	58216.000	1.117512295	21463	39650	19730	38074.000	1.087835783

IDR calculations and ratios between true replicates (Nt), pooled pseudoreplicates (Np), self-pseudoreplicates 1 (N1), and self-pseudoreplicates 2 (N2).

**Online Table 5:** Super-enhancer-associated transcription factor comparisons.

ADRN SE	Cell Lines using ROSE	Cell Lines using LILY	Cell Lines using Both
ASCL1	COG-N-415, SK-N-FI	COG-N-415, LA-N-5, NB-1643, SK-N-BE(2)C, SK-N-FI	COG-N-415, LA-N-5, NB-1643, SK-N-BE(2)C, SK-N-FI
DACH1		SK-N-AS, SK-N-FI	SK-N-AS, SK-N-FI
EYA1			
GATA2			
GATA3	COG-N-415, KELLY, NB-69, NB-1643, NB-LS, NGP, SK-N-AS, SK-N-BE(2)C, SK-N-FI	COG-N-415, KELLY, LA-N-5, NB-69, NB-1643, NB-LS, NGP, SK-N-AS, SK-N-BE(2)C, SK-N-FI, SK-N-SH	COG-N-415, KELLY, LA-N-5, NB-69, NB-1643, NB-LS, NGP, SK-N-AS, SK-N-BE(2)C, SK-N-FI
HAND1	COG-N-415, KELLY, LA-N-5, NB-69, NB-1643, NB-LS, NGP, SK-N-AS, SK-N-BE(2)C, SK-N-FI	COG-N-415, KELLY, LA-N-5, NB-69, NB-1643, NB-LS, NGP, SK-N-AS, SK-N-BE(2)C, SK-N-FI	COG-N-415, KELLY, LA-N-5, NB-69, NB-1643, NB-LS, NGP, SK-N-AS, SK-N-BE(2)C, SK-N-FI
HEY1			
ISL1	SK-N-FI		SK-N-FI
KLF13	COG-N-415, KELLY, LA-N-5, NB-69, NB-1643, NB-LS, NGP, SK-N-AS, SK-N-BE(2)C, SK-N-FI	COG-N-415, KELLY, LA-N-5, NB-69, NB-1643, NB-LS, NGP, SK-N-AS, SK-N-BE(2)C, SK-N-FI	COG-N-415, KELLY, LA-N-5, NB-69, NB-1643, NB-LS, NGP, SK-N-AS, SK-N-BE(2)C, SK-N-FI
KLF7	NB-69, SK-N-AS, SK-N-BE(2)C	COG-N-415, LA-N-5, NB-69, NB-1643, NGP, SK-N-AS, SK-N-BE(2)C	COG-N-415, LA-N-5, NB-69, NB-1643, NGP, SK-N-AS, SK-N-BE(2)C
PBX3		SK-N-FI	SK-N-FI
PHOX2A	COG-N-415, KELLY, LA-N-5, NB-69, NB-1643, NB-LS, NGP, SK-N-BE(2)C, SK-N-FI	COG-N-415, KELLY, LA-N-5, NB-69, NB-1643, NB-LS, SK-N-BE(2)C, SK-N-FI, SK-N-SH	COG-N-415, KELLY, LA-N-5, NB-69, NB-1643, NB-LS, NGP, SK-N-BE(2)C, SK-N-FI
PHOX2B	COG-N-415, KELLY, LA-N-5, NB-LS, SK-N-AS, SK-N-FI	COG-N-415, KELLY, LA-N-5, NB-1643, NB-LS, NGP, SK-N-BE(2)C, SK-N-FI	COG-N-415, KELLY, LA-N-5, NB-69, NB-1643, NB-LS, NGP, SK-N-AS, SK-N-BE(2)C, SK-N-FI
SATB1		COG-N-415, LA-N-5	COG-N-415, LA-N-5
SIX3	NGP, SK-N-BE(2)C		NGP, SK-N-BE(2)C
SOX11	NGP	LA-N-5	LA-N-5, NGP
TFAP2B	COG-N-415, KELLY, LA-N-5, NB-69, NB-LS, SK-N-FI	COG-N-415, KELLY, LA-N-5, NB-69, NB-1643, NB-LS, SK-N-BE(2)C, SK-N-FI	COG-N-415, KELLY, LA-N-5, NB-69, NB-1643, NB-LS, SK-N-BE(2)C, SK-N-FI
ZNF536	LA-N-5, NB-69, NB-1643, NGP	LA-N-5, NB-69, NB-1643, NGP	LA-N-5, NB-69, NB-1643, NGP
**MES SE**	**Cell Lines using ROSE**	**Cell Lines using LILY**	**Cell Lines using Both**
AEBP1		SK-N-SH	SK-N-SH
CBFB			
CREG1			
DCAF6			
EGR3			
ELK4			
ID1			
IFI16			
MAML2			
MEOX1			
MEOX2			
NOTCH2			
PRRX1	SK-N-SH	SK-N-SH	SK-N-SH
SIX1	SK-N-SH	SK-N-SH	SK-N-SH
SIX4			
SMAD3	LA-N-5, SK-N-AS, SK-N-SH	LA-N-5, SK-N-AS, SK-N-SH	LA-N-5, SK-N-AS, SK-N-SH
SOX9			
WWTR1			
ZFP36L1			
ZNF217	KELLY, SK-N-AS, SK-N-FI, SK-N-SH		KELLY, SK-N-AS, SK-N-FI, SK-N-SH

Adrenergic and mesenchymal lineage-specific SEs defined by Gronigen, et. al. and the neuroblastoma cell lines listed per gene in which SEs were called.
