# Supplementary material for: Epigenomic profiling of neuroblastoma cell lines
Source: Sci Data. 2020 Apr 14;7:116. doi: 10.1038/s41597-020-0458-y (PMC7156688; doi:10.1038/s41597-020-0458-y)
Supplement: Supplementary file 1 — Supplemental Figures 1 and 2 [file 41597_2020_458_MOESM1_ESM.pdf]

Table of Contents

Supplemental Figure 1……………………………………………Page 2
Supplemental Figure 2……………………………………………Page 3

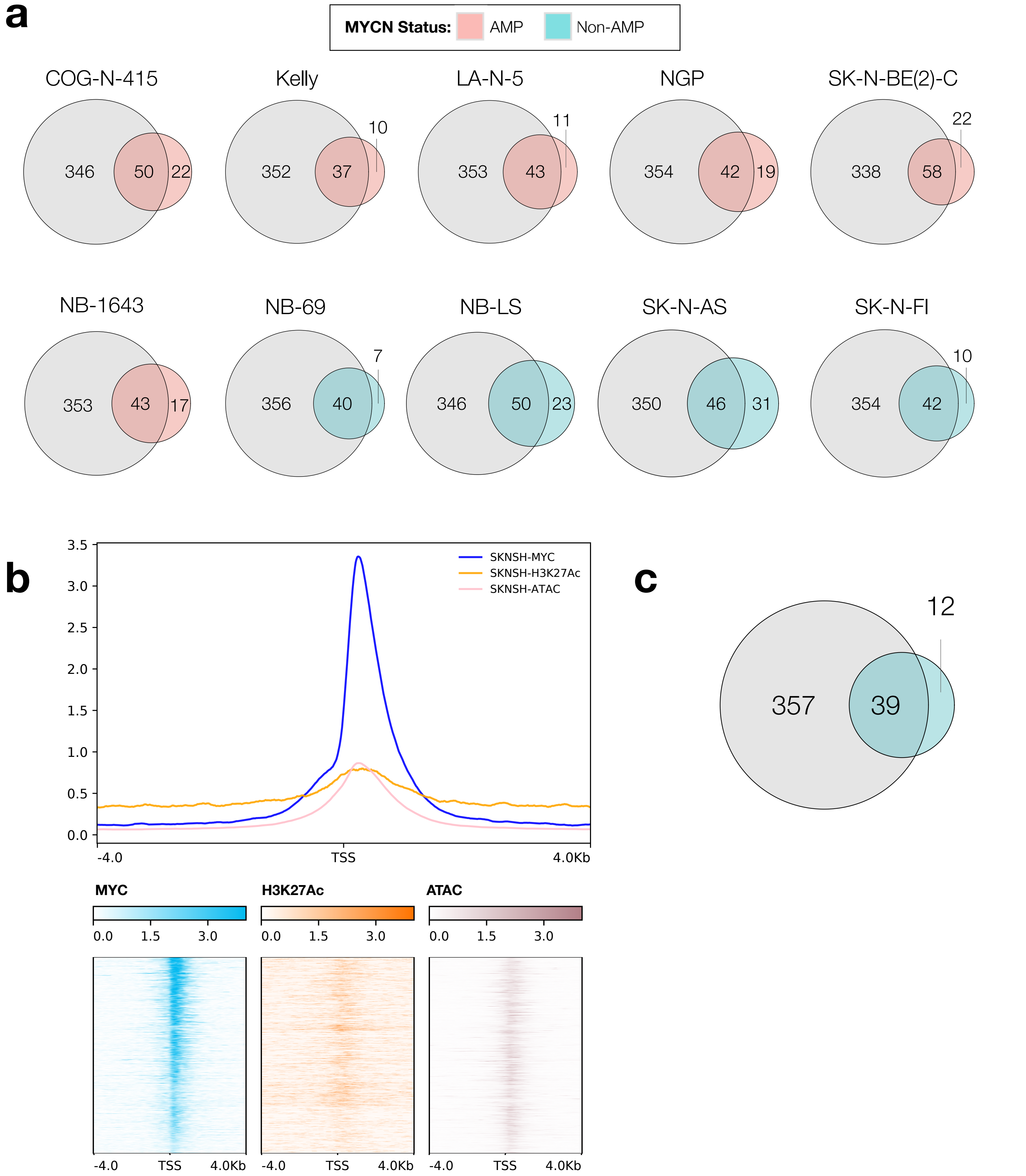

Supplemental Figure 1. Literature comparison of neuroblastoma super-enhancer calls and integration of ENCODE SK-N-SH H3K27Ac ChIP-Seq with our dataset. Venn diagrams of TF-specific SEs called with LILY using the H3K27Ac ChIP-Seq data collected in ten neuroblastoma cell lines show (a) 59-85% concordance with Boeva et al. 2017. Depicted are heatmaps and the binding profiles (b) for SK-N-SH MYC, H3K27Ac (ENCODE), and ATAC-Seq for the top 5,000 MYC peaks. (c) SK-N-SH H3K27Ac LILY super-enhancer calls were 76% concordant with Boeva, et. al, 2017 (N = 39/51).

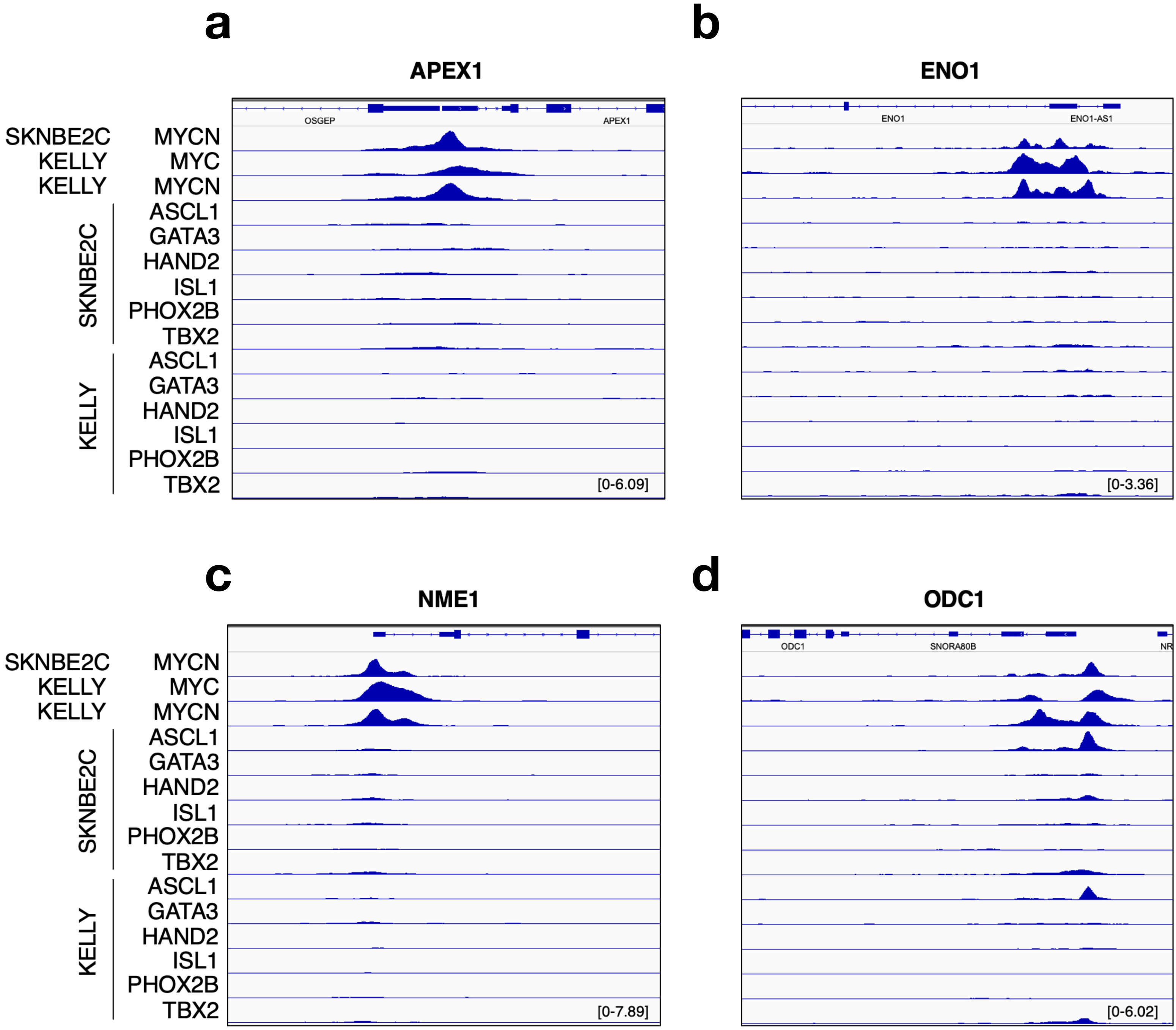

Supplemental Figure 2. Specificity of MYCN ChIP-sequencing data compared to other transcription factors. ChIP-sequencing tracks for neuroblastoma core regulatory circuit, c-MYC, and MYCN transcription factors in KELLY and SKNBE2C cell lines at (**a**) APEX1, (**b**) ENO1, (**c**) NME1, and (**d**) ODC1 loci.
